# Breaking androgen receptor addiction of prostate cancer by targeting different functional domains in the treatment of advanced disease

**DOI:** 10.1016/j.tranon.2021.101115

**Published:** 2021-05-13

**Authors:** Zoe R Maylin, Radu CB Nicolescu, Hardev Pandha, Mohammad Asim

**Affiliations:** a Department of Clinical & Experimental Medicine, University of Surrey, UK; b Department of Chemistry, University of Cambridge, Cambridge, UK

## Abstract

In the last decade, treatment for castration-resistant prostate cancer has changed markedly, impacting symptom control and longevity for patients. However, a large proportion of cases progress despite androgen deprivation therapy and chemotherapy, while still being fit enough for several more lines of treatment. Overstimulation of the androgen receptor (AR) activity is the main driver of this cancer. Targeting biological functions of the AR or its co-regulators has proven very effective in this disease and led to the development of several highly effective drugs targeting the AR signalling axis. Drugs such as enzalutamide demonstrated that the improvement in anti-tumour efficacy is closely correlated with an affinity for the AR and its activity and have established the paradigm that AR remains activity in aggressive disease. However, as importantly, key insights into mechanisms of resistance are guiding the development of the next generation of AR-targeted drugs. This review outlines the historical development of these highly specific agents, their mechanism of action in the context of defective AR activity, and explores the potential for the upcoming next-generation AR inhibitors (ARI) for prostate cancer by targeting the alternative domains of AR, rather than by the conventional ligand-binding domain approach. There is huge potential in these approaches to develop new drugs with high clinical activity and further improve the outlook for patients.

## Highlights

• This reviews endeavours to provide an up to date understanding of the androgen receptor (AR) inhibitors.
• It provides an account of the mechanism of resistance that surround AR mutations.
• Inhibitors of various targetable domains with the AR and their mechanism of inhibition provided.

## Introduction

Prostate cancer (PCa) is the most common cancer in men in the United Kingdom. It continues to be a highly significant issue and health burden affecting one in every six men during their lifetime, with around 50,000 new cases of the disease per year in total [1]. This disease is also accountable in transgender women and gender-nonconforming people, collectively making it the fourth most common cancer in the world [2,3]. PCa is a complex disease, with a variety of clinical phenotypes and unpredictable treatment responses [4]. Early detection and treatment of organ-confined disease are associated with excellent outcomes, but treatments for advanced metastatic disease are still largely palliative despite access to targeted therapies including those directed at patients with specific genomic alterations [5,6]. Primary PCa is most commonly derived from luminal epithelial cells and is characterised by reliance on the androgen receptor (AR) signalling, however rare basal cell derived PCa shows low AR expression [7,8]. We endeavour to provide a thorough account of the mechanisms underlying AR activation in PCa and outline current and upcoming AR inhibitors (ARIs) that target different AR driver domains for the treatment of aggressive PCa with a focus on patients who develop castration-resistant prostate cancer (CRPC).

## The continued reliance of castration-resistant prostate cancer (CRPC) on AR signalling: an opportunity for novel AR inhibitors

### Transcriptional activation of AR in aggressive PCa

**Fig. 1:**
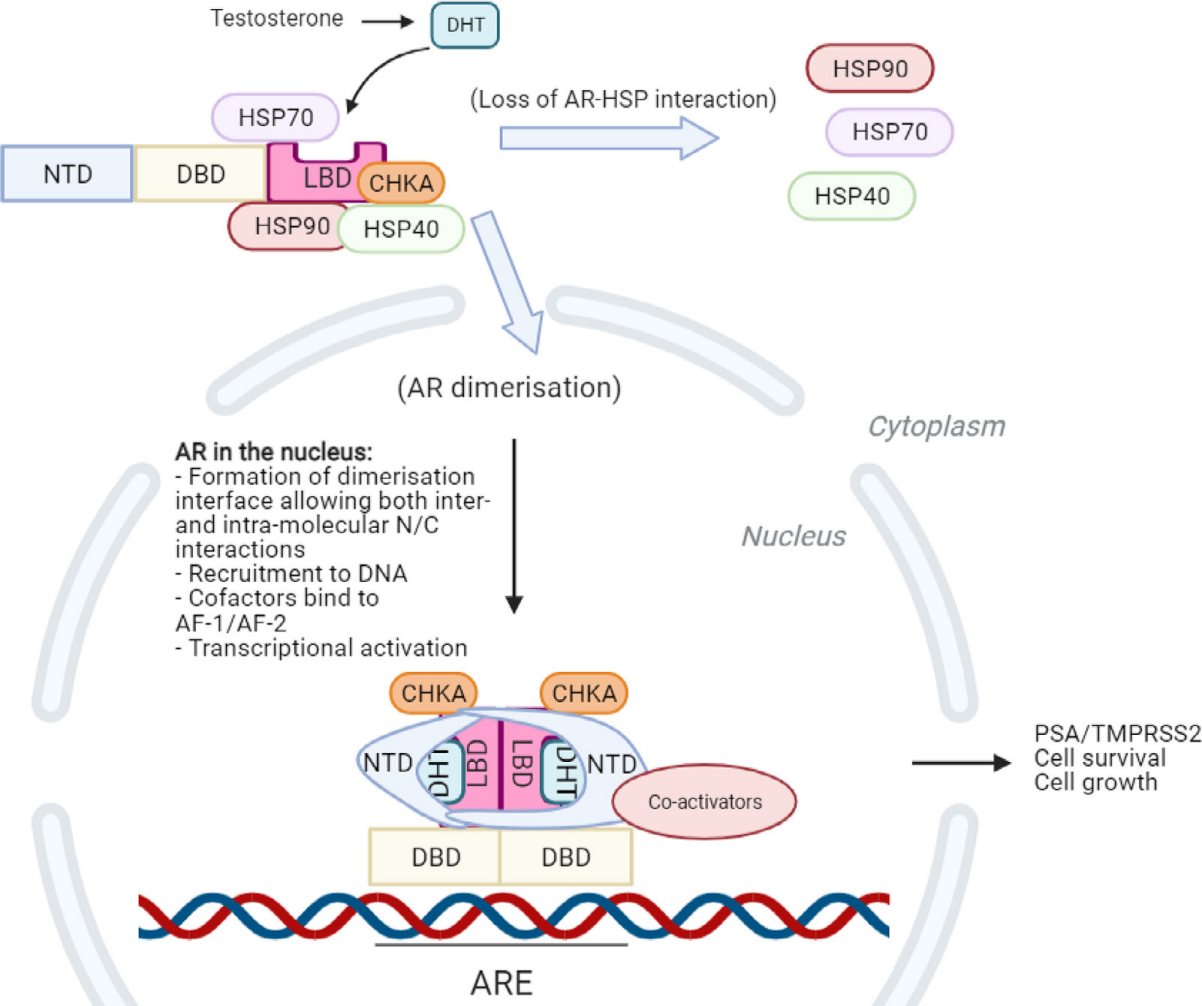
The androgen receptor (AR) signalling pathway. The AR protein is structurally made up of three main functional domains: The N-terminal domain (NTD), the DNA-binding domain (DBD) and the ligand-binding domain (LBD). A small hinge region lying between DBD and LBD is not shown. In its inactive form, the AR is bound to chaperone proteins of the Heat-shock protein (HSP) class. In addition to HSPs, Choline kinase alpha (CHKA) has recently been identified as an AR chaperone, bound both in the cytosol and the nucleus. Dihydrotestosterone (DHT) is synthesised from testosterone locally and binds the LBD of the AR, specifically the ligand-binding pocket. Upon binding, a conformation change in the AR occurs, where the HSP chaperones dissociate and the NTD and LBD form interactions (N/C interaction). Nuclear translocation occurs due to conformation change-induced exposure of a nuclear localisation signal (NLS) within the hinge region. Subsequent interactions of the NLS with the nuclear import proteins drive the AR into the nucleus. Inside the nucleus, the AR can exist in both dimer and monomer forms, however when binding to androgen response elements (AREs) of the DNA, the AR almost always exists as a dimer. Androgen-bound transcriptionally active AR dimers assume parallel “head-to-head” and “tail-to-tail” conformations, wherein the NTD, DBD and LBD all form the dimerisation interface. The two NTDs within the AR dimer adopt different conformations and surround the LBDs, with one NTD mainly interacting with its own LBD through intra-molecular N/C interaction, while the second NTD appears to form both intra- and intermolecular N/C interactions by interacting with both LBDs within the AR dimer. Transcriptional initiation can then occur by interactions with coactivators and subsequently transcription of AR target genes by other cofactor enzymes. These genes increase proliferation and survival signals to stimulate cell growth [15].

**Fig. 2:**
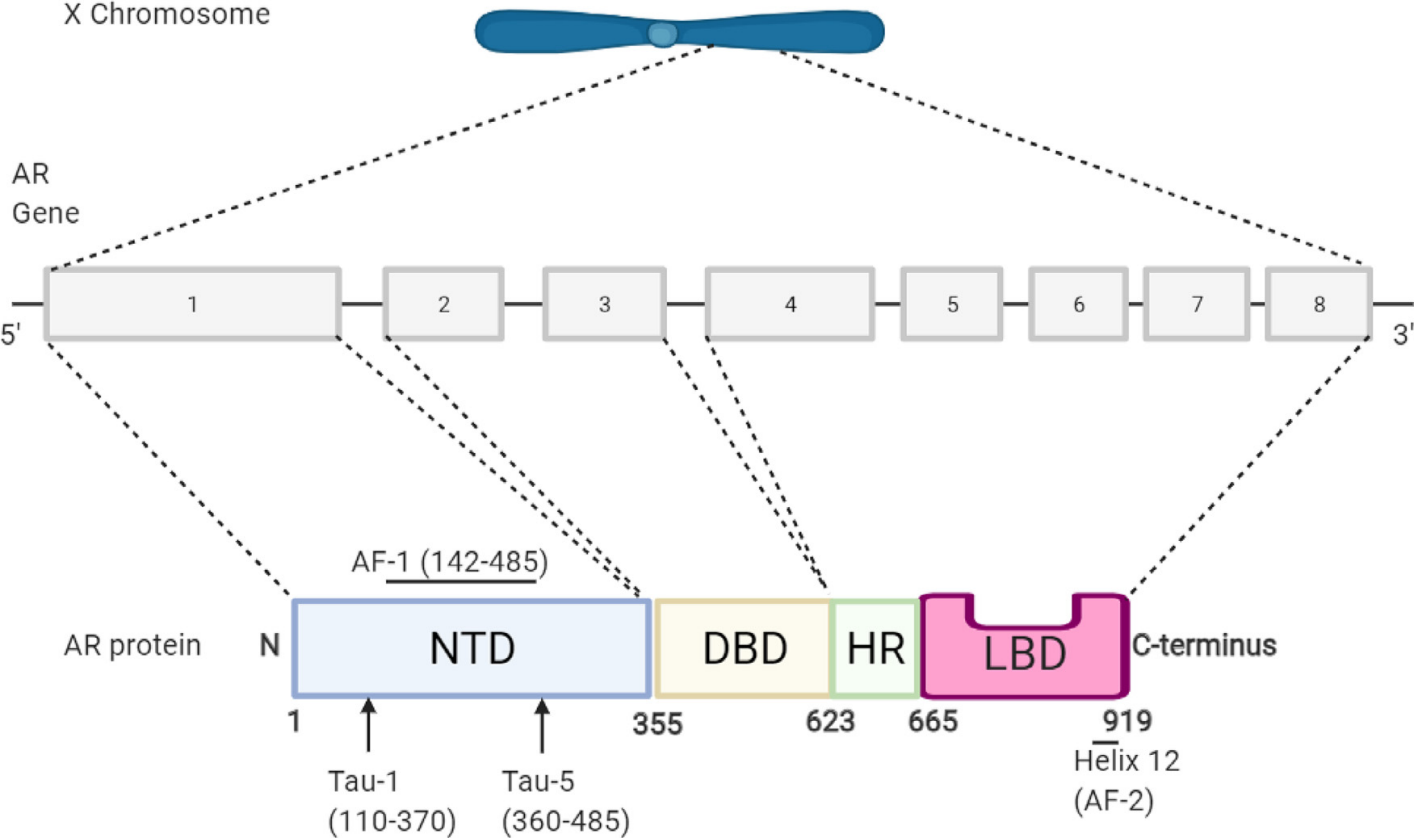
The AR gene/protein structure. The Androgen receptor gene resides on the X chromosome with 8 exons coding for the protein. Exon 1 codes for the N-terminal domain (NTD), exons 2 & 3 code for the DNA-binding domain (DBD) and exons 4-8 coding for the hinge region (HR) and the ligand-binding domain (LBD). The HR harbours the canonical Nuclear Localisation Signal (NLS) which direct the AR into the nucleus upon ligand binding. The DBD contains the P-Box recognition helix (577-581) and the D-Box site (596-600) that regulate specificity to DNA and receptor dimerisation respectively. Activation Function-1 (AF-1) resides within the NTD, with the activation domains TAU-1 and 5, while Activation Function-2 (AF-2) resides mainly within the 12^th^ and final helix of the LBD [14].

The main driver of the aggressive disease is the AR and its overstimulated, often constitutively active oncogenic signalling (Fig. 1). Normal AR signalling promotes the development and maintenance of the male reproductive system and, has a wider role in other biological processes such as in the cardiovascular and neural systems [9]. The AR is a nuclear hormone receptor that acts as a transcription factor upon activation driving the oncogenic gene expression programme to support tumour progression [10,11]. Consequently, overstimulation of the AR signalling axis can trigger uncontrolled cell growth enabling oncogenic transformation and tumour growth, therefore making the AR a major therapeutic target [12]. AR stability and function is maintained through its interaction with transcriptional cofactors (such as coactivators, corepressors), and chaperones; recently, we have discovered a novel AR chaperone choline kinase alpha (CHKA) that appears to stabilise the AR not only in the cytosol but also in the nucleus- a unique feature for an AR chaperone. The chaperone function of CHKA is independent of its kinase function and its overexpression is associated with PCa progression [13]. The AR shares homology in its protein structure with other nuclear hormone receptors where they consist of three functionally well-defined domains viz. a well-defined DNA-binding domain (DBD) within the centre of the protein structure, a ligand-binding domain (LBD) at the carboxy-terminal end of the protein, and an amino-terminal domain (NTD) (Fig. 2) [14,15].

### AR protein structure reveals several activation points

**Fig. 3:**
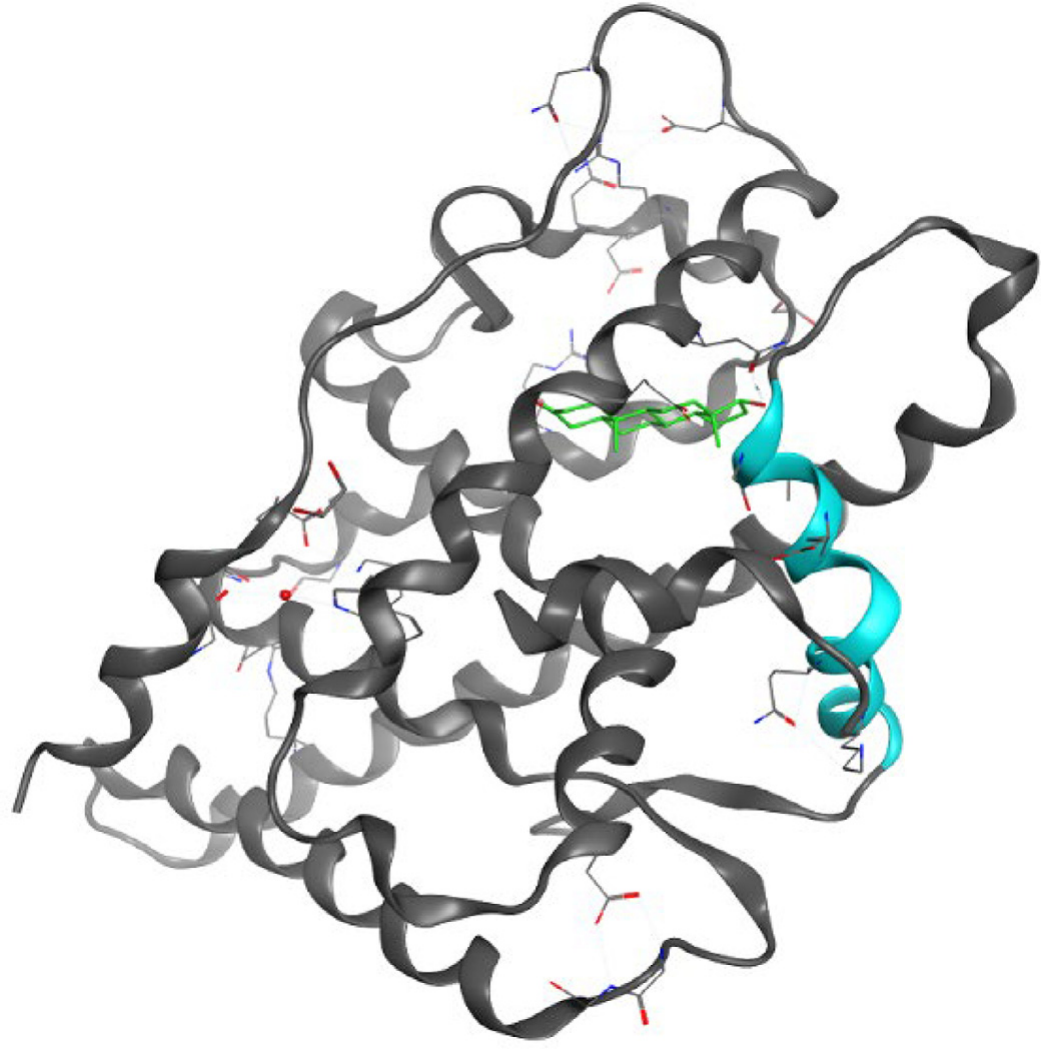
Ribbon diagram of the AR LBD with dihydrotestosterone (green) and a small peptide (FxxLF - not shown here- to mimic the N/C interaction). Helix 12 is coloured in cyan. (PDB:1T7M).

Three-dimensional crystal structures of the LBD and DBD of the AR protein have been solved, however the structure of the NTD is unknown, it is recognised to be intrinsically disordered, with its size and sequence varying greatly between nuclear hormone receptors. The NTD is primarily responsible for transcriptional activation functions and harbours activation function-1 (AF-1), which can be activated independently of the LBD. Within the AF-1 are two transactivation units (TAU), TAU-1 and TAU-5 that drive AR-dependent transcriptional activation. The DBD allows the AR to interact with DNA. It contains P-box and D-box structures that have two zinc finger motifs required for a stable interaction with specific DNA sites known as androgen response elements (AREs) within the chromatin. The DBD is highly conserved across nuclear hormone receptors, where alignment studies suggest a high overlap (at least 76%) between the AR-DBD and those of other nuclear hormone receptors [16,17]. Androgenic ligand binding to the LBD induces a conformational change and consequent dissociation of the AR from heat shock proteins in the cytoplasm allowing translocation to the nucleus [14]. In this conformational change, helix 12 repositions itself over the 11 helices that make up the ligand-binding pocket to reveal the AF-2 surface that can interact with transcriptional coactivators to activate gene transcription (Fig. 3) [18]. The LBD of the AR has up until recently been the main target for drug discovery, where blocking ligand binding can stop the receptor activation and consequently repress AR-dependent transcription.

### Patient treatment pathway: AR targeting and the emergence of CRPC

Patients will follow a certain treatment pathway as the disease progresses. If the tumour is localised at diagnosis, surgical intervention is commonly used to remove the tumour mass or patients may be treated with radiation therapy to destroy the tumour, which may be more suitable for frailer patients. High intensity ultrasound (HIFU) and cryotherapy are also options. Patients with advanced disease extending outside of the prostatic capsule are often treated with androgen deprivation therapy (ADT) employing castration by either surgery [19] or hormone therapy, aimed at decreasing the AR activation. Among other pathways that the hormone therapy targets, ARIs are given either alongside or after ADT to antagonise the AR transcriptional activity, therefore, further blunting the oncogenic effects of the AR signalling.

While this hormone blockade is initially effective, many patients eventually progress to ARIs non-responsive disease termed castration-resistant prostate cancer (CRPC) characterised by individual or a combination of rising serum PSA levels and symptomatic/radiographic progression such as the formation of bone metastases. Despite the failure of ARI, most CRPC still relies on AR signalling as evident from the success of the next-generation of ARIs [20]. Eventually, however, treatment fails due to the emergence of various resistance mechanisms that could include neuroendocrine transformation [21], and patients will be considered for participation in clinical trials of novel agents or symptom control palliation measures depending on their fitness.

## Blunting AR activity by ARIs in CRPC

ARIs that target the LBD are the predominant form of anti-androgen therapy given to CRPC patients. They are competitive inhibitors of androgens that occupy the ligand-binding pocket and therefore block androgens from binding and activating the AR. X-ray crystal structures of AR-antagonist complexes suggest displacement of the 12^th^ Helical structure occurs within the LBD, and thus the AF-2 site is distorted. Consequently, interferences with transcriptional co-factor binding can occur, leading to transcriptional inhibition [22]. This in turn will reduce downstream AR oncogenic activity eventually causing tumour to regression.

## First-generation LBD-targeting ARIs

### Steroidal inhibitors

**Fig. 4:**
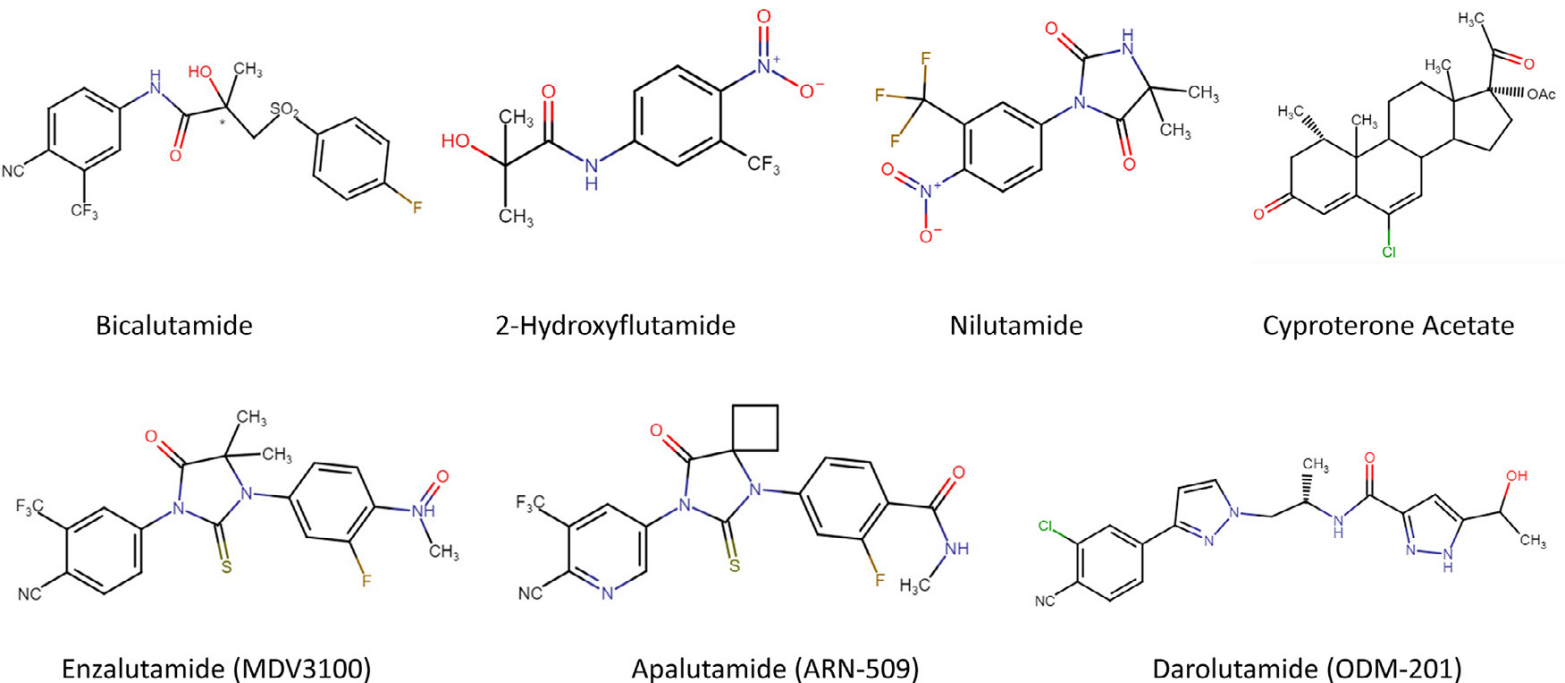
*Chemical structures of ARIs for prostate cancer treatment. These inhibitors bind the ligand-binding domain of the AR to inhibit the AR-dependent transcription. The upper panel shows first-generation ARIs, steroidal (cyproterone acetate) and non-steroidal (Bicalutamide, 2-Hydroxyflutamide, Nilutamide). The lower panel shows second-generation non-steroidal ARIs. The bulky benzene ring is common in these structures to effectively fill the ligand-binding domain of the AR [**32**,**49**,**50**,**51**,**43**,**35**,**52**].*

Steroidal ARIs such as cyproterone acetate (CPA) (Fig. 4, upper panel) were the first drugs to be used as ARIs. As well as competitively inhibiting the AR-LBD, steroidal ARIs can decrease circulating testosterone levels by inhibiting the negative diencephalic pituitary testicular system which reduces the release of the luteinising hormone leading to suppression of testosterone-producing Leydig cells in the testes [23,24]. This, therefore, reduces free androgens in the blood, further inhibiting AR activation. However, the use of CPA has been phased out due to the availability of more potent non-steroidal ARIs with increased specificity and favourable pharmacokinetics.

### Non-steroidal inhibitors

Drugs such as Bicalutamide (also known as Casodex), Nilutamide and Flutamide (Hydroxyflutamide is its active form) are first-generation, non-steroidal ARIs and competitive inhibitors of the AR (Fig. 4, upper panel). Bicalutamide widely replaced other first-generation ARIs in the clinic due to its greater potency, increased AR binding affinity and safety. Bicalutamide demonstrated IC_50_ values approximately four-times and two-times lower than Hydroxyflutamide and Nilutamide respectively [25,26]. Similarly, it has two- to four- times higher binding affinity with the AR than Hydroxyflutamide and around two times greater than Nilutamide [27], [28], [29]. Moreover, Bicalutamide administration is linked with lower incidences of hepatotoxicity as it has a longer half-life than Nilutamide and Hydroxyflutamide, the frequency of administration at relatively lower doses is reduced [30]. High doses of Bicalutamide are however well tolerated in the clinic, which allows the drug to out-compete the natural ligands of the AR, testosterone and dihydrotestosterone (DHT), although the binding affinity of Bicalutamide is around 60-times lower than that of DHT [30,29]. Furthermore, Hydroxyflutamide and Nilutamide unlike Bicalutamide have undesirable partial AR agonist activities that can lead to AR activation when administered at higher concentrations which can be potentially detrimental for patients [31].

Lack of cross-reactivity of these drugs towards other nuclear hormone receptor is also a factor to consider for treatment. Bicalutamide is known to have a low binding affinity to the progesterone receptor as an antagonist, whereas Hydroxyflutamide and Nilutamide are selective for the AR. Likewise, the inhibitory activity of the androgen-synthesising enzyme CYP17A1 can be beneficial as it reduces circulating androgen levels in the blood and has been displayed by Hydroxyflutamide and Nilutamide whereas, Bicalutamide does not interact with these enzymes [32,27].

## Second-generation LBD-targeting ARIs

### Enzalutamide, Apalutamide and Darolutamide

Second-generation ARIs such as Enzalutamide (MDV3100), Apalutamide (ARN-509) and Darolutamide (ODM-201) (Fig. 4, lower panel) are more potent than first-generation inhibitors as they display greater binding affinity for the AR, less off-target effects and have additional novel mechanisms of blocking AR action. As well as blocking the binding of androgens to the LBD of the AR, these drugs inhibit nuclear translocation of the AR whilst also impairing its chromatin binding [33], [34], [35].

Enzalutamide showed such palpable results in the phase III AFFIRM trial, that the trial was stopped early since interim analysis determined that it was able to prolong survival of CRPC patients for five months compared to placebo. This trial determined the efficacy of Enzalutamide and safety in patients who had castrate levels of testosterone, failed with chemotherapy and had a progressive disease [36]. Furthermore, the STRIVE trial highlighted the superiority of second-generation ARIs in patients with CRPC where median progression-free survival was 5.7 months for Bicalutamide but 19.4 months for Enzalutamide [37]. The PROSPER and SPARTAN clinical trials proved Enzalutamide and Apalutamide were the first drugs to show clinical benefits to CRPC patients without metastasis as well as patients with metastatic disease. It was reported that Enzalutamide and Apalutamide prolong metastasis-free survival by around 2 years compared to a placebo [38,39]. However, the administration of these drugs as part of combination therapy with other drugs (for example with ADT) should be carefully considered since they can induce several cytochrome P450 enzymes (CYPs, drug metabolising enzymes). Enzalutamide is known to induce CYP2C8, CYP2C9, CYP2C19 and CYP3A4, while Apalutamide can induce CYP3A4, CYP2C19 and CYP2C9 and therefore co-administration of drugs that also target these specific CYPs should be avoided [40,41].

A new ARI Darolutamide was developed more recently to combat resistance mechanisms and other clinical manifestations that patients encountered with Enzalutamide and Apalutamide [35] but is not yet used as the standard-of-care in the clinic with only recent FDA approval in 2019. The ARAMIS trial showed similar results to Enzalutamide and Apalutamide in terms of metastasis-free survival [42] but mechanistically, Darolutamide binds the LBD with higher affinity and has proved the more potent in terms of its interaction with W742 (Tryptophan amino acid in position 741) within the ligand-binding pocket of the AR LBD. This is suggested to be due to Darolutamide possessing the greatest flexibility in its chemical structure due to the isopropylamine linker that allows the maintenance of Van der Waals interactions with the leucine side chain, whereas Enzalutamide and Apalutamide contain a more rigid imidazolidine ring [43].

A common problem with several previous ARIs is that the drugs can cross the blood-brain-barrier and inhibit the GABA_A_ receptors in the brain causing seizures and other central nervous system issues in some patients. Darolutamide has a low brain:plasma ratio *in vivo* and therefore is much less likely to cross the blood-brain-barrier [44,45]. Furthermore, during a Phase II trial, Darolutamide showed no dose-limiting toxic effects and did not pose as a CYP-inhibitor and thus reducing the chance of drug-drug interactions and therefore may allow safer treatment of the patient [46].

### Abiraterone and its derivative D4A

LBD-targeting ARIs dominate currently in the clinic intending to reduce AR signalling and thus curbing disease progression, but other approaches are also being exploited to curb the mechanisms of resistance to ARIs. Although not a direct ARI, Abiraterone acetate is commonly used in the clinic as a second-generation drug to reduce androgen levels in the blood, with Abiraterone being its active metabolite. Clinical trials involving Abiraterone administration in combination with second-generation LBD-antagonists or with ADT has also been assessed [51,52]. Abiraterone irreversibly and selectively inhibits the CYP17A1 enzyme, an adrenal gland enzyme that catalyses the conversion of pregnenolone and progesterone to androgen precursors of testosterone, DHEA and androstenedione, which are ligands for the AR [53,54]. It does this by dual inhibition of the CYP17A1’s hydroxylase and lyase activity. Abiraterone is often administered in combination with prednisone since the Abiraterone-induced CYP17 blockade causes an elevation in mineralocorticoid levels, which are repressed by prednisone via inhibition of the adrenocorticotrophic hormone thus decreasing adverse effects [53,55]. A Phase III trial of Abiraterone demonstrated improved radiographic progression-free survival alongside a significantly delayed clinical decline in the need for initiation of chemotherapy [55]. Abiraterone contains a Δ^5^, 3β-hydroxyl moiety, which is also present in some testosterone derivatives (DHEA, A5diol), and therefore it is metabolised by the enzyme 3βHSD to Δ^5^A (D4A) [56]. D4A exhibits broader inhibition of steroidogenic enzymes than Abiraterone while it additionally directly inhibits both the full-length and mutant AR [56]. Furthermore, D4A also inhibits CYP17A1, 3βHSD and SRD5A (also enzymes for DHT synthesis) at significantly lower IC_50_ values vs Abiraterone. This therefore reduces ligand-activated AR signalling whilst also competitively antagonising the AR with comparable potency to Enzalutamide. D4A contains a 3-keto structure, similar to testosterone and DHT that allows for a modest affinity to the AR. These properties suggest that D4A is more potent than Abiraterone *in vitro* and *in vivo* and has potential as a promising clinical agent [56].

## Failure of LBD-targeting ARI therapy

**Fig. 5:**
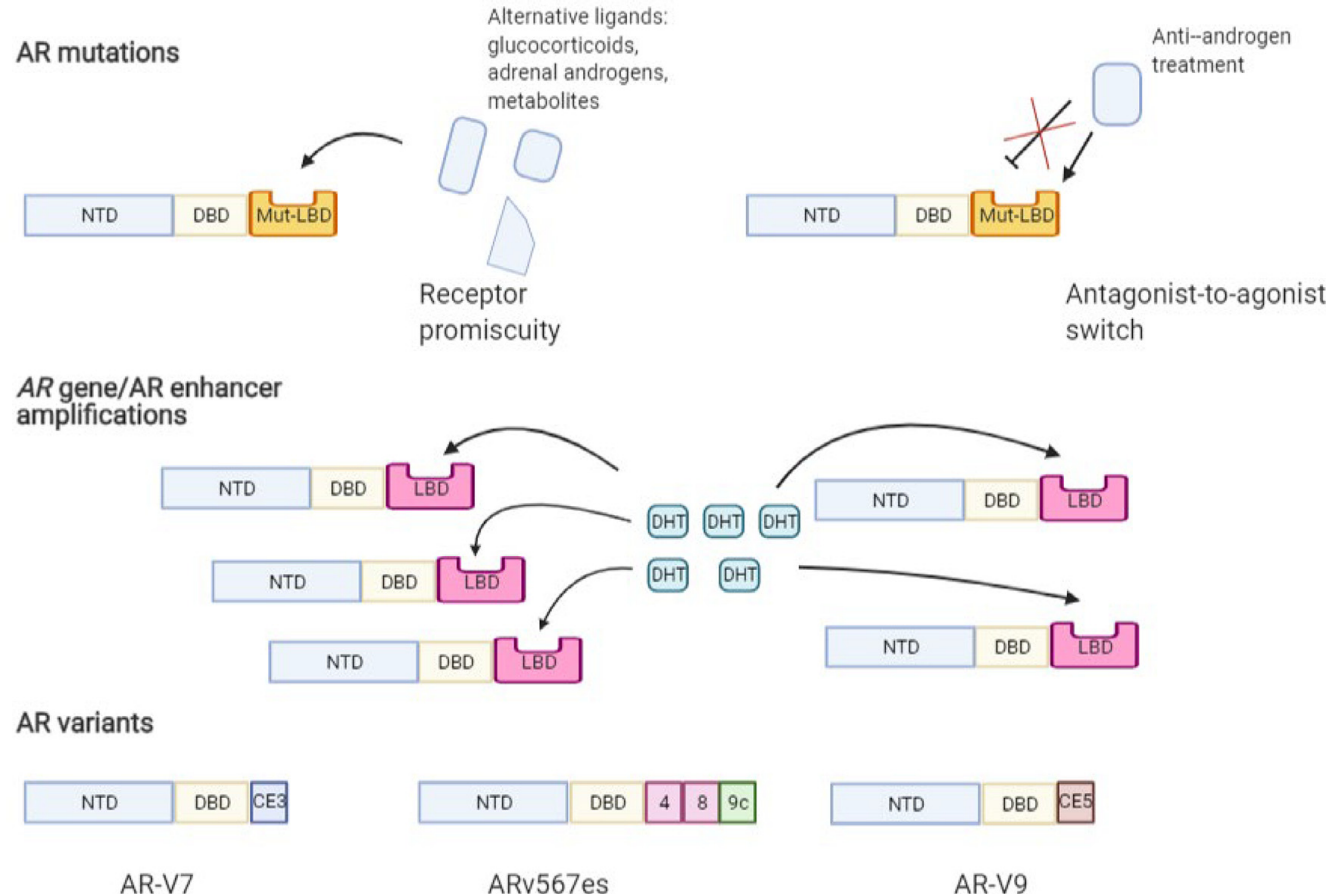
Cis mechanisms underlying ar gene activation and resistance to treatment. AR point mutations in the ligand-binding domain (Mut-LBD) cause a conformational change in the protein which can then bind to alternative ligands such as glucocorticoids, adrenal androgens and metabolites (receptor promiscuity). Contrastingly, the mutation can cause ARIs to fail by causing an antagonist-to-agonist switch, where the change in conformation allows the LBD -targeting ARIs to bind but to activate growth and survival pathways instead of suppressing. AR gene or enhancer amplifications can occur, where increased numbers of AR protein molecules are synthesised due to a greater copy number of the AR gene or hyperactivation of enhancer-induced gene expression. Consequently, more DHT can bind the receptor thus causing the growth and survival of cells to increase. AR variants (AR-Vs) can be synthesised by alternative splicing and/or AR gene rearrangements where variants can lose some or all the LBD. Without the LBD, receptors cannot be activated by ligands but can become constitutively active, allowing the signalling of the AR to occur continuously *[**59**]*. All variants shown contain the NTD and DBD (coded for by exons 1,2 & 3), however, exons 4-8 that code for the LBD are partially omitted or replaced by cryptic exons (CE). AR-V7 is coded by exons 1/2/3/CE3, Arv567es (also known as AR-V12) is coded by exons 1/2/3/4/8/9c and AR-V9 is coded by exons 1/2/3/CE5 *[*[58], [59], [60]*]*.

Following prolonged treatment with ARIs, oncogenic mutations in AR gene emerge allowing the AR to become activated despite ARIs based therapies. These “cis” resistance mechanisms such as AR LBD point mutations, AR gene/enhancer amplification and the formation of AR variants are summarised in Fig. 5, where changes to the *AR* allow for the continued and overstimulated firing of the oncogenic AR signalling pathway.

## Point mutations in AR LBD can lead to promiscuous AR activation

**Fig. 6:**
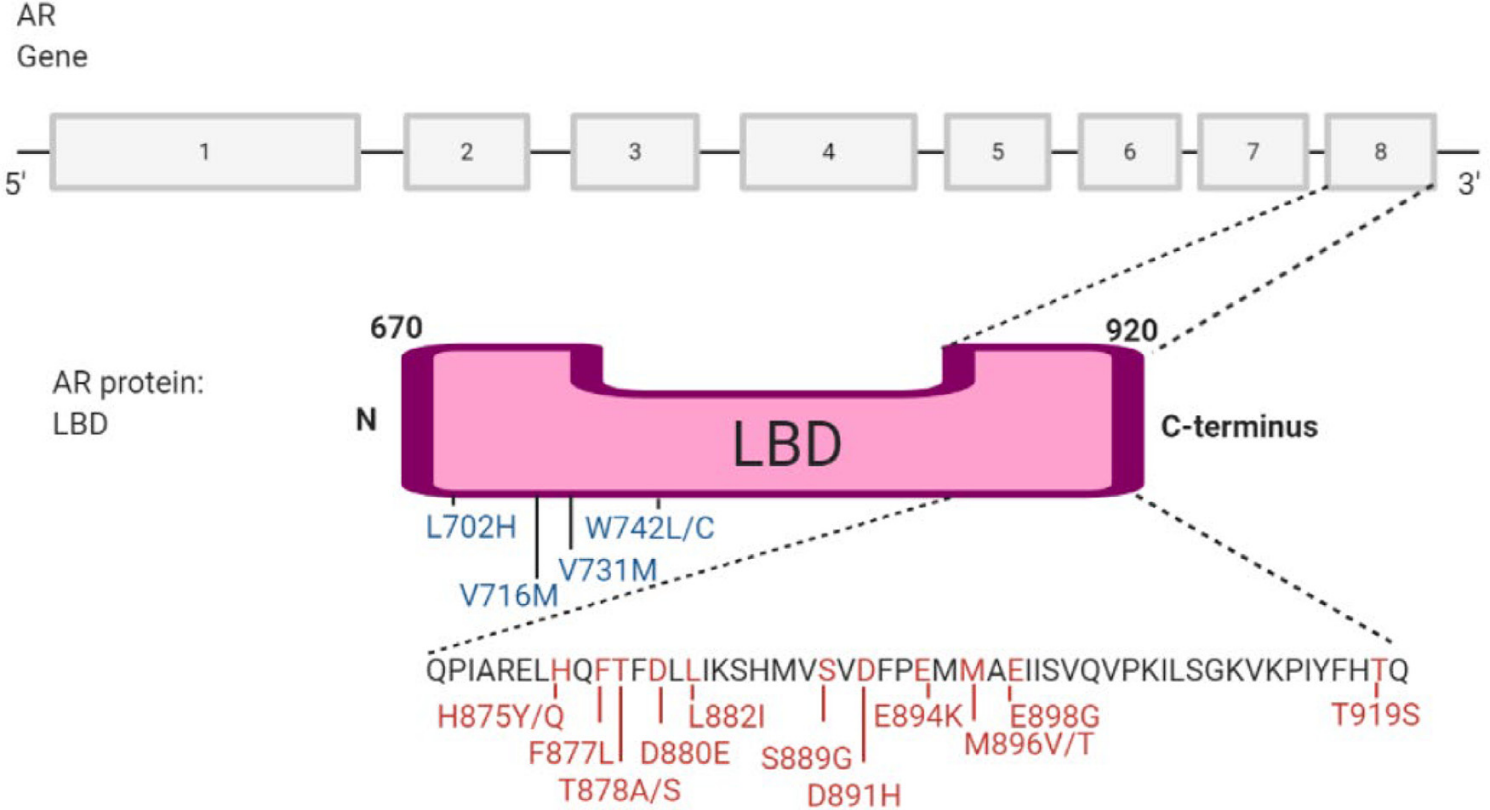
Androgen receptor (AR) ligand-binding domain (LBD) point mutations identified in CRPC patients. The gene encoding the AR has 8 exons, with most of the point mutations occurring in the 8^th^ exon (shown in red). A few LBD point mutations occur in other exons and are shown in blue. The point mutations cause a change in amino acids when the gene is transcribed and subsequently translated for example L702H indicates that leucine (L) at position 702 changes to histidine (H). These mutations allow for the LBD to change in conformation and/or activity, thus allowing for changes in binding ligands or inhibitors *[**65**]*.

Point mutations within the LBD of the AR often emerge due to treatment-induced selection pressure and are therefore uncommon in treatment-naive patients (Fig. 6). Nucleotide base pairs are substituted for others in the *AR* gene sequence and therefore codons corresponding to amino acids can change [61]. In this case, often amino acids are replaced by another that has a smaller, less bulky side chain. This creates a larger binding pocket for ligands and can remove vital interactions such as hydrogen bonds [62] causing ARI binding the AR to adapt an agonist conformation with the LBD as opposed to an ARI, whereby a bulkier molecule is needed to maintain the antagonism.

An example of this would be the mutation W742L, where the amino acid, tryptophan is changed to leucine at position 742 (this has also been seen with a change to the amino acid, cysteine [64]). This causes the AR to become promiscuously activated by Bicalutamide, instigating an antagonist-to-agonist switch. When Bicalutamide acts as an antagonist for the wild-type AR, it forms a bulky complex using the W742 and a sulfonyl linker. However, the L742 point mutation causes the B ring of Bicalutamide to shift toward the L742, which increases space between helix-12 and the drug, facilitating an agonist conformation by allowing helix-12 to move closer to the LBD [65].

Similarly, AR LBD point mutations such as F877L, and T878A (present in LNCaP cell line [66,67]) mediate the switch from antagonist-to-agonist for Enzalutamide/Apalutamide and Hydroxyflutamide/Bicalutamide treatment respectively [67], 66, 68, 63]. Clinically, these point mutations have been found in circulating cell-free DNA of patients and are considered common gene aberrations in patients undergoing Enzalutamide or Apalutamide treatment [69,63]. Studies have shown that with the T878A mutant, Hydroxyflutamide gained full agonistic properties, whilst losing its antagonistic activity entirely. Similarly, Bicalutamide treatment in T878A mutants showed a full agonist response [63]. Contrastingly, the F877L mutant will only cause weak partial agonism by Enzalutamide and Apalutamide, with around 10% and 14% of AR activation induced by DHT. However, these ARIs have shown to retain some antagonistic activity against the F877L mutant on DHT-activated AR. Interestingly, the F877L mutation causes Enzalutamide to bind the AR six times more efficiently than the wild-type receptor [63]. When the *AR* simultaneously harbours two point mutations within its LBD, it is called a double mutant. These double mutant ARs have the increased ability to cause the antagonist-to-agonist switch. For example, the F877L/T878A double mutant AR has shown to reduce Enzalutamide and Apalutamide's antagonistic activity while further increasing agonism *in vitro* by approximately an additional 20% compared to single mutants [65,67].

Mechanistically, the substitution of phenylalanine to leucine in the F877L mutation reduces steric hindrance with Enzalutamide. This is due to leucine implementing a rotamer position (a structural change in side-chain positioning) since its side chain is more flexible than the side chain of phenylalanine and thus the LBD can form an agonist conformation with Enzalutamide [67]. There is also a loss of steric hindrance with Enzalutamide with the T878A mutation in a similar fashion and a double mutant contains even less steric clashes which means Enzalutamide agonism is greater [67]. Tumours with the T878A and F877L *AR* mutations are shown to be sensitive to Darolutamide treatment and therefore Darolutamide may be a potential treatment option against such resistance mechanism [35]. Similarly, Darolutamide has been demonstrated to maintain antagonistic activity in the presence of a W742C mutation, through a hydrogen interaction between the cysteine SH group and the alcohol functionality of Darolutamide demonstrating efficacy [43].

The treatment-emergent point mutations can also lead to AR promiscuity whereby the AR is activated by other non-androgenic ligands (Fig. 5). The T878A mutation in the LBD is a classic example of a point mutation that allows AR activation by progesterone, oestrogen and adrenal androgens [70], while the double mutant L702H/T878A AR showed promiscuous activation by glucocorticoids and a 98% loss of affinity for DHT [71]. This loss of specificity may allow the AR to lose its reliance on androgenic ligands allowing constitutive activation by other non-canonical ligands, making it challenging to stop AR function in CRPC. These point mutations reduce treatment efficacy and treatment options for the patient, whereby androgen signalling is maintained or more greatly stimulated. This allows for the disease to progress quicker since inhibition of the AR is stunted and thus target genes promoting growth and survival are further activated.

## *AR* amplification

**Fig. 7:**
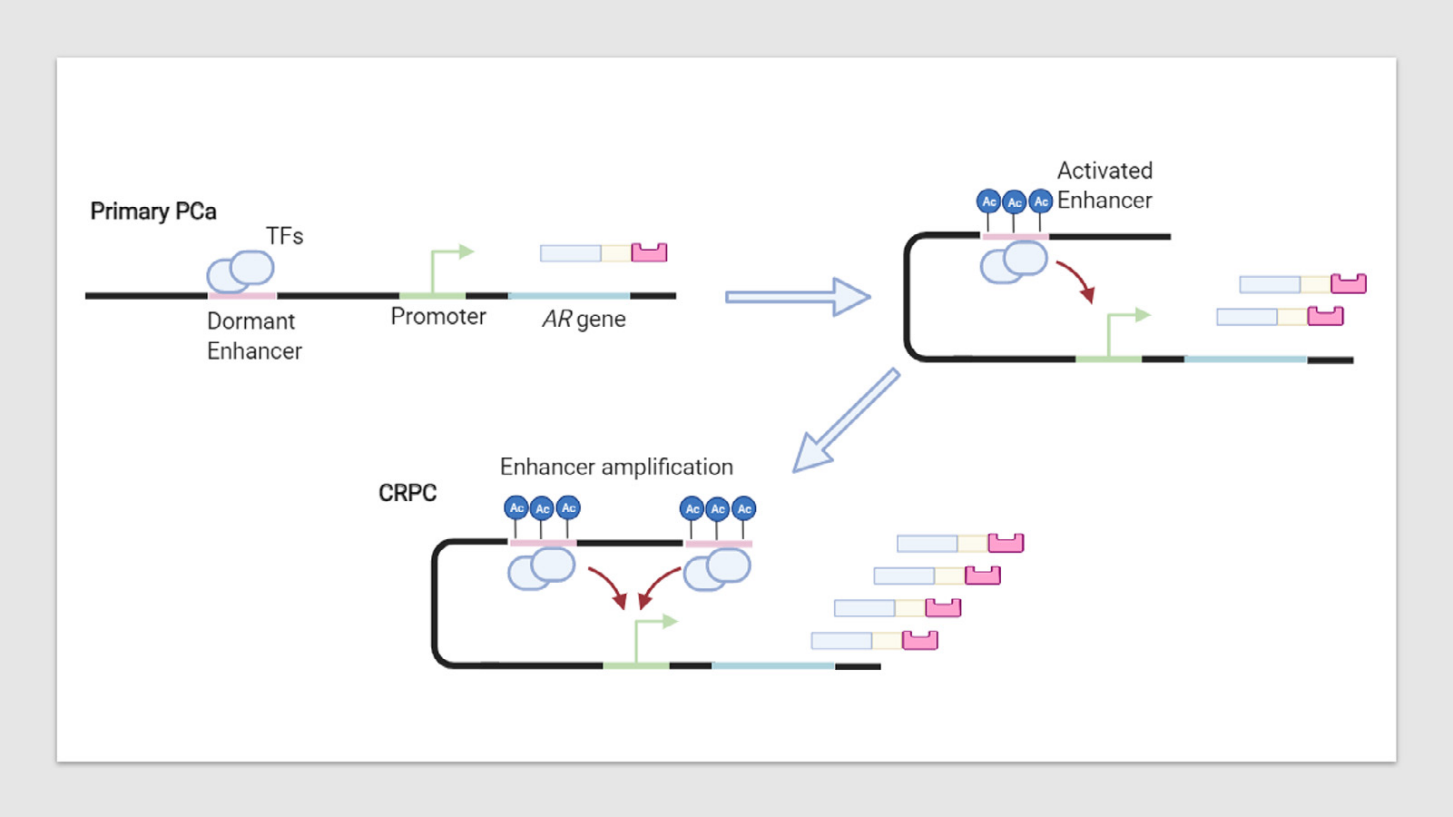
Activation and amplification of an AR enhancer drives prostate cancer progression. In primary prostate cancer, enhancers lay dormant due to histone and DNA hypomethylation or hypoacetylation. The enhancer, upstream of the AR gene locus, can be epigenetically activated by histone (H3K27) acetylation which will increase AR expression. In metastatic CRPC, this enhancer is often seen to be activated and amplified where it drives disease progression by further increasing AR expression which thus increases AR protein abundance *[**76**]*.

Comparative genomic hybridisation identified a common genetic alteration in recurrent PCa [72]. This analysis showed amplification of the chromosomal region Xq11-12, which harbours the *AR* gene. The increased copy number of the *AR* gene leads to AR overexpression and increased AR protein levels (Fig. 5). The AR protein produced from these gene amplification events is identical in structure and ligand specificity as the wild-type AR [73], however, its sheer abundance could allow some AR to escape inhibition by anti-androgens resulting in therapy failure. Additionally, a recent AR enhancer amplification mechanism (Fig. 7) in CRPC patients explained the increase in AR expression commonly observed in CRPC patients [74]. Deleting this somatically acquired enhancer resulted in decreased proliferation associated with decreased AR levels, reinforcing the role of AR gene expression in conferring ARI resistance.

Amplification of both the *AR* gene, as well as its enhancer can also create a hypersensitivity of the AR to low levels of androgens created by castration [75,74]. The greater abundance of the AR protein may lead to more receptors binding their ligand, DHT even when circulating concentrations of DHT are low. This could lead to the greater firing of the androgen signalling pathway causing disease progression [76]. Both *AR* gene amplification as well as somatically acquired AR enhancer events are extremely uncommon in hormone-naive prostate cancer, however, they are present in a large subset of CRPC cell lines and tissues [77], which indicates that treatment-induced selection pressure allows for this adaptive genomic response [73], 76, 74]. A study found prostate tumours that had a mean *AR* gene copy number of 2.7-28 per cell, as opposed to the intended one copy dramatically increased AR protein level [73]. Treatment of PCa tumours containing the amplified AR with AR antagonists such as Enzalutamide, Apalutamide and Darolutamide may be ineffective as, although the drugs are still specific to the AR, they may not be able to antagonise all AR proteins [78,79]. Increasing treatment doses could aid this issue, however, it could equally increase the treatment pressure, which in turn could enable the development of other resistance mechanisms.

## AR variants

Following treatment of PCa with LBD targeting anti-androgenic drugs, AR variants (AR-Vs) have emerged that can circumvent AR inhibition by conventional LBD-targeting ARIs. These AR-Vs lack some or all the LBD, with carboxy-terminal extensions replacing the LBD that are encoded by unique transcripts (Fig. 5). The NTD and DBD usually remain the same as in the wild-type AR. Since these AR-Vs lack the LBD, they bypass the requirement of agonistic ligands for activation, thus becoming constitutively active. Growing evidence suggest AR-Vs are oncogenic and can initiate transcription without a ligand thus, constantly driving AR activity that further promotes cell growth and survival [80,59]. This can therefore accelerate tumour growth whilst increasing the resistance to AR-LBD targeting therapies. The oncogenic potential of variant, AR-V7 was further demonstrated by a study showing that combined AR-FL and AR-V7 degradation by inhibitors of a de-ubiquitinase, USP7, reduced the proliferative activity of PCa cells [81], thus advocating the role of AR-Vs in cancer progression.

Over 20 AR variants (AR-Vs) have been identified to co-express with wild-type AR in human prostate cancer cell lines, xenografts or clinical specimens [60]. AR-Vs have been detected in normal prostate and treatment-naive prostate cancer, however, they are much more common in CRPC, leading to the hypothesis that AR-Vs are created in higher quantities due to treatment pressure [58]. These variants cannot be targeted with LBD-targeting drugs to reduce the AR activity since they do not contain the LBD and therefore AR-targeted treatment options for patients dramatically diminish. AR-V7 is the most common of the constitutively active variants, with AR-V567es and AR-V3 also in this category. Other variants such as AR-V9 tend to be conditionally active, with its activity dependent on the cellular context [82,57].

**Fig. 8:**
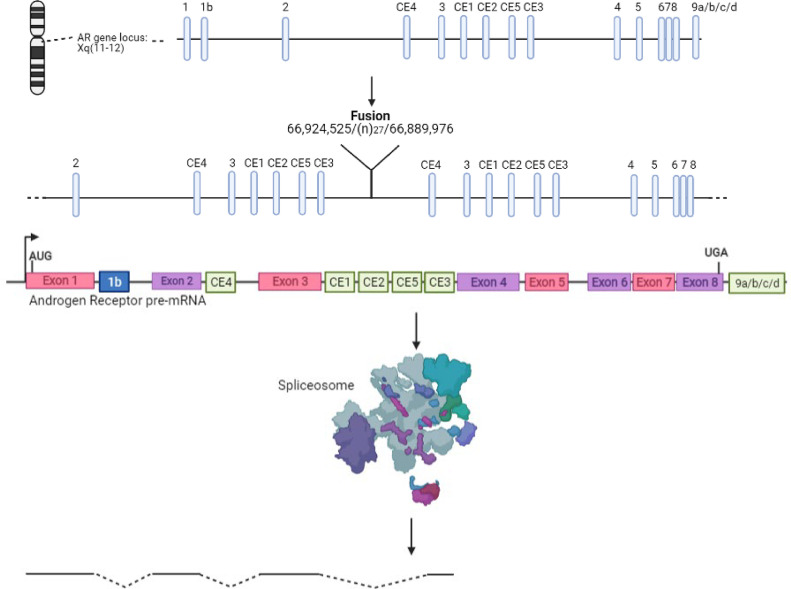
The generation of Androgen Receptor-Variants (AR-Vs) can be caused either by a combination of intragenic rearrangements and alternative splicing or alternative splicing alone with examples shown. a) Intragenic rearrangements are seen in the prostate cancer cell line CWR22Rv1 cause tandem duplication of AR cryptic exons resulting in a greater abundance of cryptic exons leading to the increased likelihood of their preservation in alternative splicing. The fusion occurs at the 66,924,525^th^ base pair of the first AR gene and the 66,889,976^th^ base pair of the second AR gene, with the fusion also containing a de novo 27 base pair sequence. b) Alternative splicing of AR pre-mRNA results in the formation of a truncated AR variant, AR-V7. Exons 1, 2, 3 and cryptic exon 3 are translated to form AR-V7, a constitutively active form of the AR [[60],[86]]. Conventional exons shown in pink and purple while cryptic exons shown in green. Exon 1b expressed only when alternative promoter used.

These truncated AR-Vs form due to alternative splicing and/or structural gene rearrangements of the *AR*. Alternative splicing occurs such that a different pre-mRNA sequence is included in the mature mRNA compared to the intended sequences (Fig. 2) due to differential excision of specific exonic sequences [58]. The result is that an altered combination of exons is translated and thus a protein with an altered structure is produced, lacking a functional LBD. For example, AR-V7 mRNA is spliced at the alternative 3’ splice site next to a cryptic exon (CE), CE3, as opposed to the 3’ splice site by exon 4, this, therefore translates into a C-terminal truncated form of the AR protein (Fig. 8) [58,82]. Alternative splicing of the *AR* gene is much more predominant in androgen-driven prostate tumours that have undergone anti-androgen therapy. Interestingly, the recruitment of splicing factors such as ASF/SF2 and U2AF65 to 3’ splice sites is significantly higher in PCa cells exposed to Enzalutamide [58,84]. Furthermore, other studies have shown upregulation of many splicing factors or their interactors in PCa. For example, lncRNA metastasis-associated in lung adenocarcinoma transcript-1 (*MALAT1*) associates with the splicing factor SF2 but *MALAT1* is regulated by suppressive androgen response element in the promoter and therefore AR antagonism (e.g Enzalutamide treatment) will increase *MALAT1* expression and thus the SF2 splicing factor [83,85]. This could potentially lead to increase AR-Vs expression following Enzalutamide treatment.

A structural gene rearrangement of the *AR* that is commonly seen is a tandem duplication that causes the formation of variants (present in CWR22Rv1 cell line) [86]. This occurs due to an intragenic rearrangement and duplication of a genomic section (~35kb) that comprise a cluster of alternative cryptic *AR* exons and consequently synthesis of these cryptic exons is increased (Fig. 8). Enhanced splicing of these alternative exons and subsequent translation forms the truncated AR-Vs that can further drive the growth of tumour cells [86].

These AR-Vs are present in an array of cell lines, however VCaP and CWR22Rv1 are the most commonly used and both contain variants AR-V7 and AR-V3 [87], [88], [89]. These cell lines contain both full-length AR and the truncated variants and thus are much less sensitive to anti-androgen treatment and growth is not solely determined by the abundance of androgens since the AR-Vs do not contain a domain where the ligand can bind.

Interestingly, AR-Vs are almost always co-expressed with full-length AR, which has been shown to be essential for AR-V activation. While the exclusive role of AR-Vs expression in driving CRPC is yet to be completely established, PCa cell line models such as R1-X-11 and R1-D567 have been generated, expressing clinically relevant AR-Vs [90,91] and AR-Vs depletion has been shown to decrease their growth.

## Upregulation of intra-tumoral androgen synthesis

In addition to the several “cis” mechanisms that cause gain-of-function mutations in the AR gene, it is now well established that the AR pathway can be stimulated within the tumours by the upregulation of androgen synthesis. 3β hydroxysteroid dehydrogenase (3βHSD1) an AR regulated enzyme, which catalyses the formation of extragonadal androgens within the tumours from adrenal precursors thus fuelling cancer progression [**94**] Gain-of-function mutations in *3βHSD1* such as N367T has been linked to increased enzyme accumulation, leading to enhanced androgenic flux within the CRPC [**93**]. Additional point mutation in *HSD3B1* (1245A>C) have not only been linked to increased androgen synthesis but opposes Abiraterone suppression by Abiraterone, suggesting an important role of this enzyme as a key driver of CRPC progression [**94**,**95]**.

## New ARIs to combat the resistance mechanisms

Table 1 briefly summarises the novel emerging ARIs that target the AR signalling axis aiming to combat treatment resistance mechanisms. Please note that many of them supported by pre-clinical evidence, pending further studies and regulatory approval.

**Table 1:** Novel emerging AR-directed treatment options.

Category of inhibitor	Drug/group of drugs name	Mechanism
AR protein degradation enhancer: reduces the abundance of AR protein	Niclosamide	Degrades solely AR-V7 via the proteasome-dependent pathway *[**98**,**99**]*.
	ASC-J9	Degrades AR-FL and AR-V7 via a proteasome-dependent pathway, increasing interaction between AR protein and Mdm2 (E3 ubiquitin ligase) *[**100**,**101**]*.
	UT-34	Named a ‘pan AR antagonist’. Degrades AR via an unknown non-common proteasome-dependent pathway. Can also bind NTD and LBD to inhibit AR *[**102**]*.
	PROTACs	Heterobifunctional small molecule. Structurally made with AR-LBD antagonist linked with E3 ubiquitin ligase which tags AR for degradation *[**103**,**104**,**105**]*.
AR-NTD targeting: inhibits AR transcriptional activity	EPI derivatives (Anitens)	Reversibly interacts with TAU-5 within the AF-1 region of NTD *[**106**]*.
	Sintokamides	Binds AF-1 region of NTD at residues 142-485 *[**107**]*.
	QW-07	Binds unknown region of AR NTD, suppressing interactions with AREs and coactivators *[**108**]*.
	BiAb 3E10-AR441	Bispecific antibody complementary to aa 302-318 in NTD *[**109**]*.
AR-DBD targeting: inhibits AR interaction with DNA	Pyridium pamoate	Interacts with AR-DBD already bound to DNA to prevent RNA pol II recruitment *[**110**,**111**]*.
	VPC compounds	Binds region under DBD P-box reducing interactions with AR and chromatin *[**112**,**113**]*.
	Hairpin polyamides	Bind minor grooves of DNA due to specificity with AREs to disrupt cofactor associations *[**114**,**115**]*.
AR dimerisation inhibitor	VPC-17005	Binds L594-S613 of AR-DBD D-box to inhibit dimerisation *[**116**]*.

## AR degradation-enhancing agents

Degradation of proteins is a natural manifestation, often directed at misfolded or unfolded proteins, but is also vital for the maintenance of cellular function. This is usually carried out via the process of ubiquitination of the protein in question and consequent degradation by the proteasome [115,[116]. However, treatments are now being considered to modulate and enhance this process where enhanced degradation of the AR would, in principle, reduce the abundance of the AR protein and thus reduce its oncogenic signalling.

### Niclosamide

**Fig. 9:**
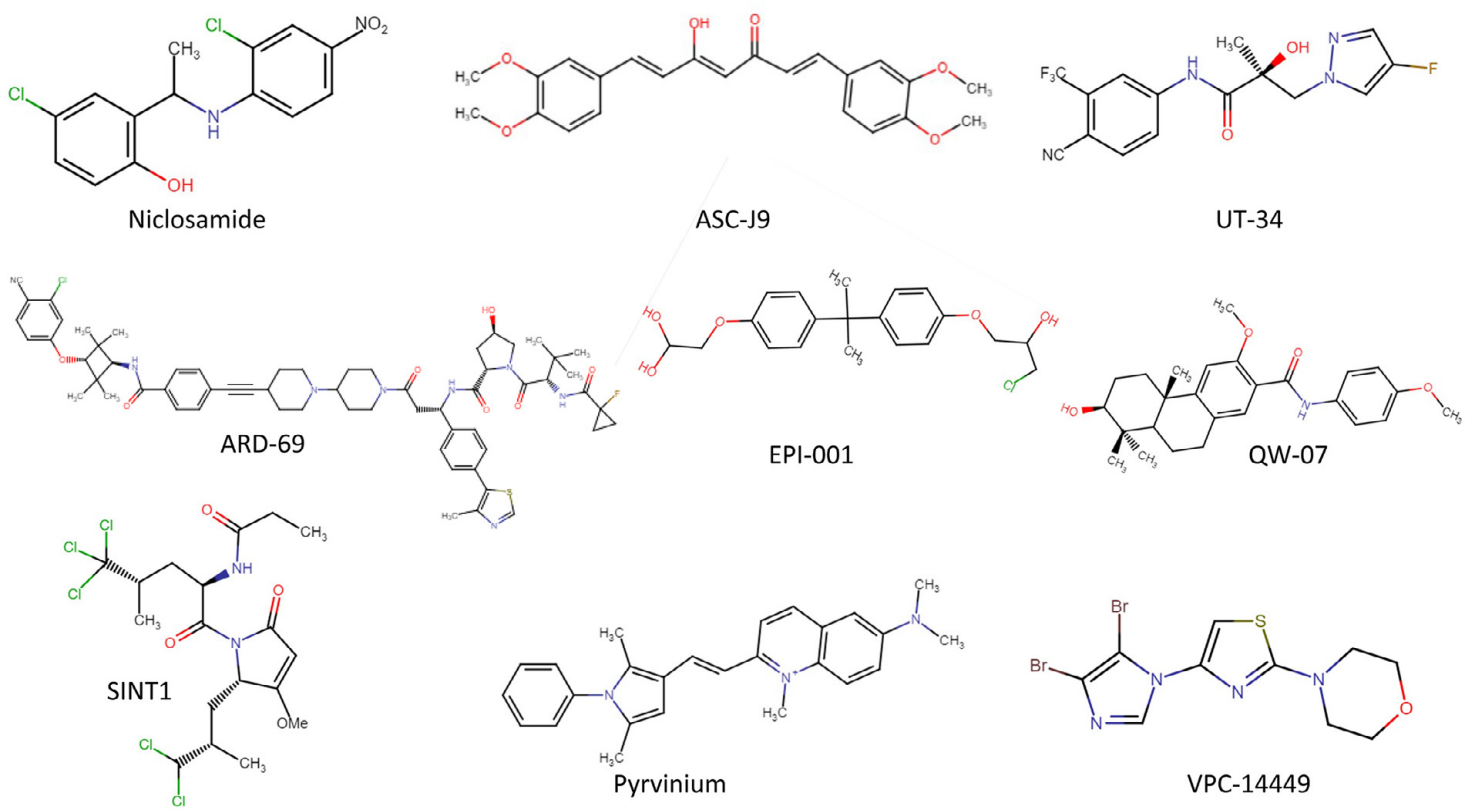
Chemical structures of AR inhibitors that do not target the ligand-binding domain. AR degraders: Niclosamide [134], ASC-J9 [135], UT-34 [100] and ARD-69 [101]. NTD-targeting agents: EPI-001 [124], SINT1 [105] and QW-07 [106]. DBD-targeting agents: Pyrivinium [108] and VPC-14449 [136]. Not all structures were available for all inhibitors discussed in the review.

Niclosamide (Fig. 9) is an FDA-approved anthelmintic drug, used to treat tapeworm infections that have recently shown to mediate solely AR-V7 protein degradation via the proteasome-dependent pathway (the exact mechanism has not been reported) [96]. Preclinical data show Niclosamide inhibits the expression of AR-V7 and lessens the resistance to LBD-targeting drugs in tumours with this variant, presumably by restoring the expression of full-length AR [96]. In fact, the combination of Enzalutamide and Niclosamide in Enzalutamide-resistant models allows for degradation of the constitutively active AR-V7, diminishing the concentration of AR protein without an LBD that Enzalutamide can bind to and inhibit and thus there is a higher percentage of full-length AR protein to be inhibited by Enzalutamide. A combination of this treatment showed inhibition of tumour cell growth and the induction of apoptosis, whilst also reducing recruitment of the AR to target gene promoter regions [96].

Two-Phase I clinical trials are currently ongoing to assess dosing and tolerability of Niclosamide treatment when in combination with Enzalutamide for CRPC patients [117]. Niclosamide has also shown to repress androgen-independent AR activation by blocking Interleukin-6 (IL-6) activated Signal transducer and activator of transcription 3 (STAT3) interaction with the AR NTD. This leads to transcriptional inhibition of the AR and is hypothesised to aid the reversal of Enzalutamide resistance [118,[119]. Niclosamide is now undergoing Phase II clinical trials for CRPC combining treatment with Abiraterone acetate and prednisone since also demonstrating palpable results with such combination therapy [97,117].

### ASC-J9

ASC-J9 or Dimethylcurcumin [120] (Fig. 9) is known as an AR degrader, previously used to topically treat acne and other conditions such as male pattern hair loss [117]. ASC-J9 has shown to reduce cell proliferation and invasion by enhancing AR protein degradation via ubiquitination, leading to suppression of tumour growth both *in vitro* and *in vivo* [98,[99]. This is achieved by increasing the interaction of the AR protein with Mdm2 (E3 ubiquitin-protein ligase) and the subsequent phosphorylation of Akt and Mdm2, key molecules critical to the proteasome-dependent pathway [99]. Studies have demonstrated this degradation encompasses both full-length AR and AR-V7 in multiple PCa cell lines [99,[121]. ASC-J9 also showed to increase apoptosis via NF-kB signalling that upregulated the expression of BCL-2-associated X protein (BAX), a protein implicated in caspase-dependent apoptosis [98,[122]. Furthermore, ASC-J9 exhibited suppression of invasion by inducing sumoylation of STAT3 to block its phosphorylation resulting in suppression of epithelial-mesenchymal transition signals that induce morphological changes in AR-independent cell lines [123].

### UT-34

UT-34 (Fig. 9) was named as a pan AR antagonist as it showed inhibition of multiple AR isoforms/mutants. The compound was able to inhibit wild-type, LBD-mutant and AR-Vs both *in vitro* and *in vivo*, reducing the proliferation of cells and growth of tumours [100]. Mechanistically, UT-34 can promote degradation of the AR via the ubiquitin-proteasome pathway, while also inhibiting AR transcriptional activity by binding to the NTD and LBD. Its true mechanism for AR degradation needs to be explored further since UT-34 does not use the common pathways of proteasomal degradation [100]. UT-34 does, however, require the interaction between TAU-5 within the AF-1 region of the NTD to establish AR degradation [100]. UT-34 treatment showed no cross-reactivity between nuclear hormone receptors and demonstrated safe and stable metabolism and pharmacokinetics [100]. This multi-inhibition could be key for the development of novel prostate cancer drugs.

### Proteolysis targeting chimaeras

Proteolysis targeting chimaeras (PROTACs) are a promising, new agent class that can be employed to degrade the AR. PROTACs are hetero-bifunctional small molecules and structurally are made up of an AR binder/antagonist joined to an E3 ubiquitin ligase ligand via a linker [101]. E3 ubiquitin ligases are enzymes involved in the proteasome pathway that tag substrates (protein) with ubiquitin for degradation. Mechanistically, the AR antagonist will bind the AR and joined by the linker, an E3 ubiquitin ligase will bind its ligand and tag the AR protein for degradation, thus reducing the abundance of the AR and dampening its activity, leading to repression of PCa progression [101], [102], [103]. ARD-69 [101], the SARM-nutlin PROTAC [102] and ARCC-4 [103] are some examples of AR targeting PROTACs and these differ in the AR antagonist used, the size and type of linker and the ligand for the different E3 ubiquitin ligases. ARD-69 (Fig. 9) treatment *in vitro* has demonstrated efficient degradation of the AR protein by 95% and can inhibit cell viability with 100 times more potency than AR LBD antagonists. Furthermore, single doses of the compound were able to reduce AR protein levels *in vivo [**103**]*.

## NTD-targeting AR inhibitors

With the NTD being critical for the transcriptional activity of the AR, inhibiting this part of the AR protein would be predicted to block AR activity regardless of the presence or absence of the LBD, and thus such inhibitors will have the potential to treat all AR-Vs since all functional AR isoforms rely on a functional NTD. It remains a challenge to create specific inhibitors for the NTD because of its intrinsically disordered nature and thus undetermined 3D structure and therefore current NTD inhibitors are often discovered using compound screening libraries.

### EPI derivatives

Epoxide based EPI-001 (Fig. 9) and its derivatives (denoted ‘Anitens’ or ralaniten analogues) were first introduced in 2010 as small molecule NTD inhibitors and have since improved in efficacy and safety [124]. *In vitro* studies have shown EPI reduces the chromatin binding of AR thus decreasing transactivation, leading to growth inhibition. Furthermore, it has also shown to block the N/C interdomain interaction of the full-length AR and disrupt protein-protein interactions of the AR with factors that aid the stabilisation of the N/C interaction. However, it does not competitively prevent ligands binding to the LBD as it solely interacts with the NTD. Moreover, it blocks interactions between the AR and transcriptional cofactors such as CREB-binding protein and RAP74 [124] whilst being a potent inhibitor of IL-6 induction of the AR NTD via STAT3 [105]. Interesting, the general thiol alkylating activity of EPI-001 has an agonistic effect on peroxisome proliferator-activated receptor-gamma (PPARγ) [125]. PPARγ plays a role in prostate maintenance and development, where it bidirectionally interacts with the AR [126], and thus its modulation by EPI-001 treatment leads to inhibition of AR transcription and a reduction in AR protein levels, suppressing PCa cell growth [125].

Data first suggested that EPI interacts with the AF-1 domain of the NTD by direct binding and creating conformational change in the AR polypeptide. Further studies then showed that EPI reversibly interacted with TAU-5, in which EPI forms a reversible complex with a precise conformation of AF-1 which allows for nucleophilic attack by a protein side-chain on the C-Cl bond of EPI, thus forming an adduct incapable of activating transcription [104]. It was later discovered that EPI binds three regions (residues 353-364, 397-407 & 433-466) of TAU-5 where RAP74 of the basal transcriptional machinery interacts and requires binding to all three to establish an effect [105].

EPI-001 showed to be non-specific to the AR, however, where it inhibited the growth of AR-negative prostate cancer cell lines as well as in a breast carcinoma cell line where it modulated both oestrogen and progesterone receptors [125]. EPI-506 was the first of its kind to be tested in humans however the clinical trial was terminated due to a high pill burden of 18 capsules a day [117]. EPI-7386, the most recent derivative entered Phase I clinical trials in 2020, where it has shown 20-fold higher anti-androgenic potency compared with EPI-001 along with greater stability. EPI-7386 has also a similar IC_50_ to Bicalutamide and Darolutamide so can be administered at similar doses. EPI-7386 has shown to be effective in prostate tumour models driven by wild-type, mutant and AR-Vs and continues to inhibit transactivation [124,[127]. EPI-7070 (another EPI analogue) and Enzalutamide have demonstrated synergistic inhibition of AR-dependent growth in an Enzalutamide-resistant cell line, showing almost total inhibition of DNA synthesis at the cell cycle S phase [128]. This suggests using the combination of both an LBD-targeting drug and an NTD-targeting drug could be the way forward for the next generation of PCa therapeutics.

### Sintokamides

Sintokamides (Fig. 9) are polychlorinated small peptides derived from the marine sponge *Dysidea* sp. family that antagonise and uniquely bind the AF-1 domain of the NTD [105]. Combination experiments with EPI-002 and sintokamide A (SINT1) showed an additive effect and therefore proposed that these two compounds bind different regions of the NTD. Experiments showed that SINT1 bound a recombinant AF-1 protein at residues 142-485, therefore it is hypothesised that SINT1 binds closer to the N-terminus of the AF-1, potentially overlapping or within TAU-1 and thus inhibiting AR transactivation [105]. SINT1 treatment demonstrated inhibition of wild type and AR-V signalling, a decrease in cell proliferation and showed tumour regression in CRPC tumours. SINT1 has shown specificity for the AR and does not interact with other steroid hormone receptors and does not bind the LBD of the AR. SINT1 does, however, have a short half-life and therefore questions about its clinical practicality have been raised [105].

### QW-07

A recent study showed QW-07 to be another compound that blocks the transcriptional activity of the NTD (Fig. 9). It can suppress transactivation in both *in vitro* and *in vivo* models that are driven by full-length AR and AR-Vs significantly better than EPI-001 [106], thus reducing cell proliferation and showed to cause CRPC tumour regression. It does this by suppressing interactions between the AR and androgen response elements of DNA and coactivators such as CBP (a bridging factor that stabilises AR binding to AREs by interacting with the AR NTD) [106]. Experiments demonstrated that it can directly bind to the NTD, but further work would need to map the precise binding site on the AR [106].

### Monoclonal antibodies targeting an AR domain

Another approach in targeting the NTD is the use of monoclonal antibodies. These antibodies are often used to bind cell surface antigens or extracellular molecules since they cannot penetrate the cell membrane and flag them for degradation by the immune system. mAb 3E10, derived from an anti-DNA autoantibody from lupus nephritis-infected mice, however, can penetrate mammalian cells rapidly and its uptake is dependent on ENT2, a nucleotide salvage receptor. Bispecific antibodies to target the NTD were developed using 3E10 as a scaffold and combining single-chain variable fragments (scFv) additions joined by linkers. AR441 (a known anti-AR antibody [129] was used as the scFv component due to its epitope being located in the NTD of AR, creating the antibody BiAb 3E10-AR441. The antibody was able to inhibit the AR transcriptional activity by binding to and inactivating the NTD of full-length AR and AR-Vs and reducing transactivation of AR target genes leading to inhibition of androgen-dependent cell growth *in vitro* [107]. Whether this antibody can penetrate cancer cells *in vivo* remains be determined.

## Compounds targeting AR-DBD

Targeting the DBD of the AR could lead to preventing the AR from docking onto DNA and/or interacting with DNA and its co-factors. The interaction between the AR and DBD occurs due to the DNA recognition α-helix in the P-box region of the DBD inserting into the major groove of DNA [110]. Inhibition of this interaction would prevent AR chromatin binding preventing it from oncogenic signals that enable the progression of cancer. Notably, it would inhibit full-length AR and AR-Vs since both isoforms contain the DBD. Theoretically, whilst point mutations in the DBD against DBD-targeting treatment can still occur, most have been found to inactivate the AR. This is because high structural and functional conservation of the DBD sequence is required for its ability to bind to the DNA and therefore resistance against DBD-targeting inhibitors will likely take longer to arise compared with LBD-targeting drugs [110]. Due to the high homology of the DBD structure with other nuclear hormone receptors, however, finding therapeutics that are solely selective for one nuclear receptor is difficult [110].

### Pyrvinium pamoate

Pyrvinium pamoate (PP) (Fig. 9), a cyanine dye derived from quinoline, was the first small-molecule inhibitor of the AR DBD to be discovered, with pyrvinium being the active component of the compound [108]. PP demonstrated a greater inhibition of AR activity than Hydroxyflutamide and Bicalutamide *in vitro* while combination treatment of PP with these LBD-inhibitors synergistically disrupted AR activity leading to decreases in AR activity and cell growth [108]. Similar results were shown *in vitro*, where PP treatment alone reduced tumour weight but a greater reduction was seen by combination therapy with an LBD-targeting inhibitor [108]. Furthermore, this inhibition was exhibited in models with both wild type and AR-Vs, which demonstrates the ability of PP to inhibit clinically relevant constitutively active AR variants.

Further results suggest that PP only interacts with the AR DBD once the AR is bound to DNA. While PP does not stop the AR nuclear localisation or binding to DNA, it can prevent RNA polymerase II recruitment causing transcriptional repression [109]. It was found that PP treatment changes the association between the AR and two RNA helicases, DDX5 and DDX17, known for their involvement in splicing and act as factors for the AR [109]. While PP treatment decreased AR signalling, it also reduced oestrogen and glucocorticoid signalling, suggesting a potential cross-talk with other nuclear hormone receptors suggesting that PP-based inhibition could have detrimental off-target effects [109]. However, this treatment could inhibit PCa growth in contexts where the AR function is bypassed by the oestrogen or glucocorticoid receptor [130,[131].

### VPC compounds

A group of compounds were discovered to be solely selective to the AR DBD (as opposed to other NRs) such that AR signalling is inhibited. VPC compounds (VPC-14449 being the lead inhibitor, Fig. 9) demonstrated inhibition of wild-type AR and AR-Vs in *in vitro* models of CRPC leading to suppression of growth [111]. Similarly, in an *in vivo* CRPC model, VPC-14449 treatment reduced tumour size and repressed PSA synthesis [16]. Studies have identified a pocket within the AR DBD P-box region (S759-K610) as a binding site and showed that AR residues Y594, Q592 and K593 are key in VPC binding to the AR DBD [110], [111], [16]. This binding reduced interactions between the AR (especially wild-type) and chromatin. In another PCa model driven by AR wild-type, VPC-14449 was able to block the recruitment of the AR on chromatin binding sites for its target genes (*FASN, FKBP5* and *TSC2)*, whilst it was only able to block recruitment for *FASN* and *FKBP5*, but not the *TSC2* gene in an AR-V model [111]. This suggests that the location on the AR that VPC compounds interact with may be altered slightly in AR-Vs and thus treatment may be less effective against AR-Vs. VPC treatment is less likely to cause toxic effects compared to PP since PP treatment causes Poly-ADP ribose polymerase (PARP) cleavage, whereas VPC does not [16]. VPC treatment *in vivo* however, demonstrated that a higher dose of VPC was required to elicit an effect and therefore metabolic stability needs to be further explored [111].

A few other compounds have shown to elicit inhibition of the DBD, for example, hairpin polyamides have shown to be sequence-specific to AREs and bind to minor grooves of DNA disrupting associations between transcription factors to their DNA binding sites, RNA polymerase II activity and replicative helicase activity and thus blocking AR transactivation [112,[113].

## Agents targeting AR dimerisation

AR dimerisation (Fig. 1) has been discovered to be a critical process for the activation of all forms of AR, where this complex is required for binding to ARE's within DNA [132,[133]. A minority of AR full-length mutants cannot dimerise and can only interact with high affinity ARE oligonucleotide sequences as a monomer with the transcriptional output often hampered [132]. Contrastingly, AR-V monomers haven't proven to be functional and therefore either heterodimerisation with AR-FL or another AR-V or homodimerisation is key for chromatin binding and thus transactivation [133]. It is therefore likely that targeting AR dimerisation could significantly reduce AR-dependent transcription and thus PCa progression. The D-box site within the DBD controls AR dimerisation and is where the main dimer interface is located. Depending on the orientation of the two receptors, interactions between the LBDs (specifically the co-activator grooves) can occur or between one receptor's NTD (FQNLF motif) and the other receptor's LBD [114].

### VPC-17005

Prototypical compounds that block AR dimerisation were developed as a result of virtual screening of the DBD structure, with the aim to inhibit AR transcriptional activity via an LBD-independent mechanism. VPC-17005 was found to be the lead compound in these screening assays and showed to interact within residues L594-S613 of the DBD D-box's exposed surface pocket. The binding of VPC-17005 to the DBD forms a stable ternary complex with DNA, whereby consequently chromatin-AR interactions are significantly reduced [114]. VPC-17005 treatment *in vitro* showed to reduce cell viability in both AR-FL and AR-V containing cell lines while reducing AR transactivation. Most mutations that arise within the D-box reduce AR activity e.g. A596T-S597T and therefore it is unlikely that mutations will occur due to the treatment pressure of VPC-17005. Furthermore, cross-reactivity of VPC-17005 with other nuclear hormone receptors was not observed, showing its specificity for the AR [114].

## Future perspectives and conclusions

The life expectancy of patients with advanced prostate cancer is slowly increasing, and the impact of newer ARIs has been easily evident in the clinic. However, for the majority of patients, responses are finite and almost all patients with the advanced disease succumb to their disease.

CRPC is a complex disease and mechanisms in its development and progression can be multifactorial. The molecular events underlying resistance to current drugs are similarly complex and incompletely understood. Although this review focused on the ligand-dependent resistance, ligand-independent mechanisms of resistance can also occur such as aberrant activation of oncogenic signalling increase by the exploitation of numerous pathways, such as those driven by NF-kB [137], PI3K [138], growth factor pathways [139,140] or modification of co-regulators [141]. The presence of AR-Vs is a large contributor to treatment resistance. AR-Vs in circulating tumour cells or blood samples can act as a biomarker [142], [143], [144], [145] already guiding clinicians in terms of choice of therapy.

To address the need for a better understanding of the exact mechanisms that underlie resistance to anti-androgens, there is an urgent need to evaluate clinically relevant patient tissue, procured at different time points in the evolution of the disease. Sequential biopsy tissue after each line of treatment would allow comprehensive genomic and pathway analysis and determine which proteins are interacting with the AR at specific stages in the cancer advancement. This understanding will then drive the development of further potential therapies.

**Fig. 10:**
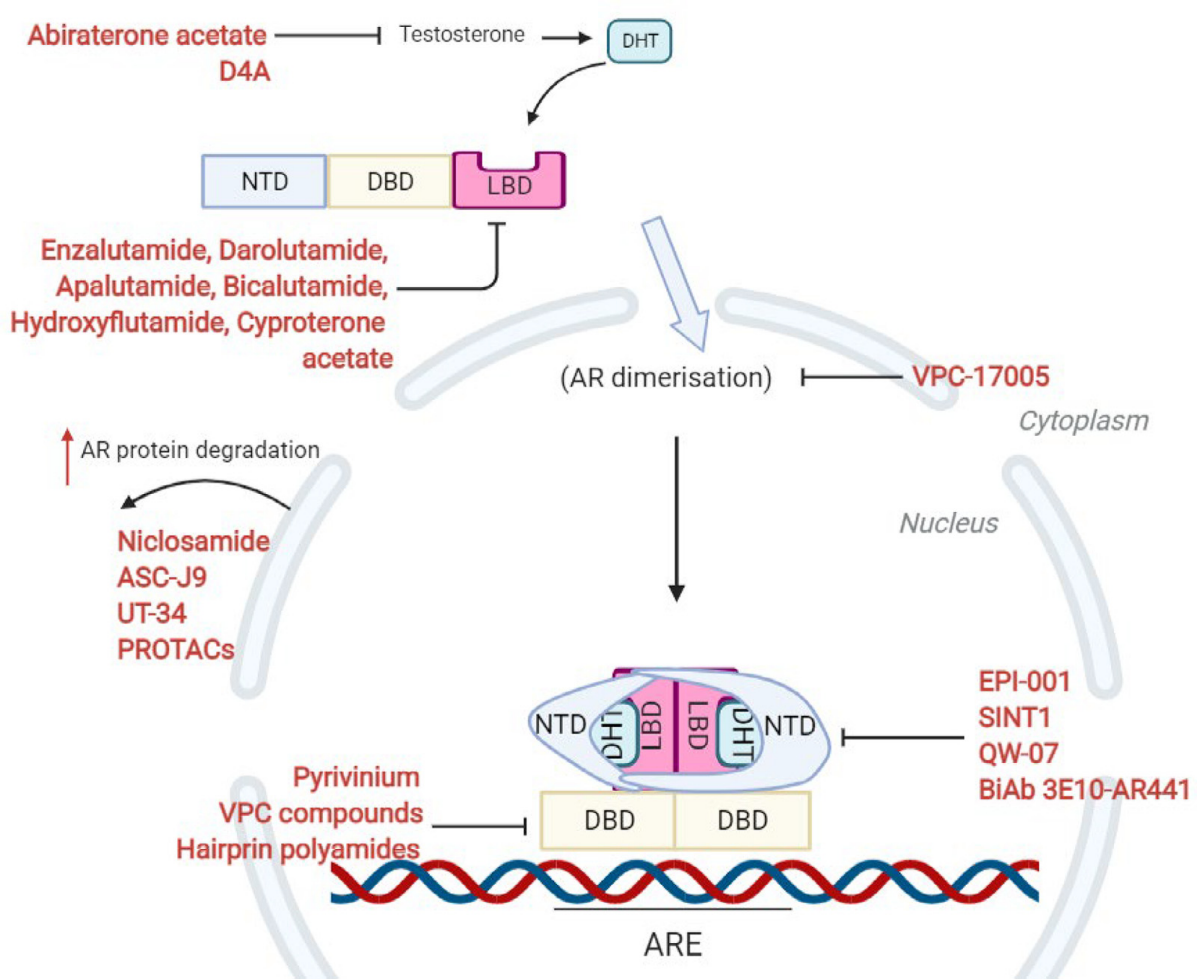
Summary of current and experimental treatments that target the AR signalling axis via different mechanisms. Current therapies such as Enzalutamide competitively inhibit the ligand-binding domain (LBD) while therapies such as Abiraterone acetate inhibit the production of dihydrotestosterone (DHT). Emerging therapies have been able to bind the N-terminal domain (NTD) of the androgen receptor and thus suppress transactivation of AR target genes, bind the DNA-binding domain (DBD) thus preventing interaction with DNA, inhibit AR dimerisation so that it cannot interact with DNA or upregulate AR protein degradation to reduce the abundance of the receptor.

Astonishing progress has been made in this field in the last ten years with the development and utilisation of second-generation anti-androgens that provide huge benefits to patients now. Darolutamide is the newest of the LBD inhibitors, shown to have equal potency to Enzalutamide but can maintain antagonism in the presence of LBD point mutations due to its bulkier structure making it a promising candidate for standard therapy. There is a great promise, however, in the novel emerging therapies discussed in this review that inhibit alternative modular domains of the AR (summarised in Fig. 10). These drugs have a huge scope for producing even more successful outcomes for patients either as monotherapy or in combination with other therapies, with the ultimate aim of reducing AR signalling unachievable by current therapies. These developments will continue to build on the platform current AR targeted drugs have achieved, and the huge contribution these drugs have made to the prognosis and wellbeing of patients with advanced prostate cancer all over the world.

## Authors’ contribution

All authors wrote the manuscript, Z.M and M.A edited it, Z.M and R.C. Bizga Nicolescu prepared figures.

## Availability of data and materials

Not applicable

## Financial support and sponsorship

This work was supported by a doctoral college, University of Surrey PhD studentship award (Z.M), and a Prostate Cancer Foundation (USA) young investigator Award (M.A).

## Ethical approval and consent to participate

Not applicable

## Consent for publication

Not applicable

## Declaration of Competing Interest

All authors declared that there are no conflicts of interest.
